# Retention of Urine in Elderly Men

**Published:** 1882-02

**Authors:** J. W. S. Gouley


### RETENTION OF URINE IN ELDERLY MEN.

(DR. J. W. S. GOULEY *in Proc. Med. Soc. of Kings.*)

The author, after speaking of the various classifications, proceeds to divide the subject into two parts. In the first, he treats of the acute cases, and in the second of the chronic. He next contrasts the the principal features of the two classes. The acute form comes on suddenly and is accompanied by pain and desire to urinate and is caused by engorgement of prostrate, spasmodic stricture of the vesical neck, congestion at site of stricture, œdema of the mucous membrane, plugging of canal by foreign bodies, such as muco-purulent plug or calculus.

The determining causes are exposure to cold or over indulgence in eating, drinking, or venery. It is preceded by dysuria, and accompanied by violent straining. It rapidly becomes complete and is followed by dribbling of urine. The distended bladder forms a prominent tumor in the hypogastric region. We may or may not have cystitis.

In the chronic form we have an insidious invasion, the retention being incomplete for weeks and months. There is loss of muscular power and sensibility in the bladder.

It results from enlarged prostate, urethral stricture, stone, tumors, follows acute retention or disease, or injury of the great nerve centers from which the bladder is supplied. It is not accompanied by pain or violent straining. We are apt to have dribbling of urine and cystitis is always present.

Chronic retention of urine from prostatic hypertrophy is next considered. May be due to unilateral, bilateral, or centric enlargement. At first a little urine is retained, this gives rise to spasmodic contracion of bladder and hypertrophy of its muscular coats, also by decomposition, it causes cystitis; on account of the frequent passage of urine and dribbling, the patient often thinks he is passing too much water.

Similar conditions may arise from the formation of muscular valvule. This may occur as a complication of stricture, stone, &c. It may follow acute retention, which has remained unrelieved for two or more days and has not received proper after-treatment.

Symptoms and progress of chronic retention. These are often so insidious as to give rise to no uneasiness in the mind of the patient. He finally consults the physician because he thinks he is passing too much urine. This is due to the fact that he suffers from dribbling of urine and very frequent micturition.

In such cases the bladder is never entirely emptied of its contents. Its walls are thickened and inflamed, the ureters dilated. The bladder often contains one or more sacculations, which vary greatly in size. These may be perforated and pericystic abscesses may result.

Diagnosis.—Chronic retention may be confounded with peritoneal dropsy, abdominal tumors, cancer of the liver or omentum, hydronephrosis, hydatids of the liver or omentum, &c., &c.

Dr. Gouley next relates a case of chronic retention that was mistaken for hydatids and tapped—nine and a half pints of urine, being drawn off. The patient died two weeks afterward from exhaustion.

Treatment of chronic retention. The surgical treatment should consist, first, in withdrawing the offending urine, second, in relieving the cystitis, third, in endeavoring to remove the obstacles to the spontaneous expulsion of urine.

When the retention is due to stricture or unsuccessful evacuation, catheterism, followed by dilatation, divulsion, or urethrotomy to or above the normal size of urethra and suitable irrigation of bladder will generally be sufficient.

Periodic catheterism is necessary to prevent recurrence. When due to supra-montaneal hypertrophy of the prostrate, systematic catheterization should be resorted to and this if begun early will often prevent the disease from giving any trouble.

Dr. Gouley next discusses the kinds of catheters to be used in these cases. He condemns all metallic, very hard non-metallic and soft catheters which are armed with a stylet.

The catheter should not be under No. 9 English and eyes should always be smooth. The Doctor gives preference to the gum catheters made by Benas' Son, of New York, or to the velvet-eyed rubber catheters of Tiemann, of New York, or Ford of Boston. An overdistended bladder should not be emptied too suddenly, but weak solution of boracic acid should be injected as urine is withdrawn.

After failing with other catheters, he has always succeeded with Mercier's invaginated catheter. Dr. Gouley does not describe the excision or incision of the prostrate which he recommends when the obstruction is due to hypertrophy of that organ.

Discussion.—Dr. J. C. Hutchinson recommends the use of a catheter 16 or 18 inches long, as ordinary catheters may be too short to reach the bladder.

